# Clinical scoring model based on age, NIHSS, and stroke-history predicts outcome 3 months after acute ischemic stroke

**DOI:** 10.3389/fneur.2022.935150

**Published:** 2022-08-05

**Authors:** Gang-yu Ding, Jian-hua Xu, Ji-hong He, Zhi-yu Nie

**Affiliations:** ^1^Department of Neurology, Jiading District Central Hospital Affiliated Shanghai University of Medicine and Health Sciences, Shanghai, China; ^2^Department of Neurology, Shanghai Tongji Hospital, Tongji University School of Medicine, Shanghai, China

**Keywords:** acute ischemic stroke, prognosis, risk factors, prediction models, nomogram

## Abstract

**Background:**

The clinical nomogram is a popular decision-making tool that can be used to predict patient outcomes, bringing benefits to clinicians and patients in clinical decision-making. This study established a simple and effective clinical prediction model to predict the 3-month prognosis of acute ischemic stroke (AIS), and based on the predicted results, improved clinical decision-making and improved patient outcomes.

**Methods:**

From 18 December 2021 to 8 January 2022, a total of 146 hospitalized patients with AIS confirmed by brain MR were collected, of which 132 eligible participants constituted a prospective study cohort. The least absolute shrinkage and selection operator (LASSO) regression was applied to a nomogram model development dataset to select features associated with poor prognosis in AIS for inclusion in the logistic regression of our risk scoring system. On this basis, the nomogram was drawn, evaluated for discriminative power, calibration, and clinical benefit, and validated internally by bootstrap. Finally, the optimal cutoff point for each independent risk factor and nomogram was calculated using the Youden index.

**Results:**

A total of 132 patients were included in this study, including 85 men and 47 women. Good outcome was found in 94 (71.212%) patients and bad outcome in 38 (28.788%) patients during the follow-up period. A total of eight (6.061%) deaths were reported over this period, of whom five (3.788%) died during hospitalization. Five factors affecting the 3-month prognosis of AIS were screened by LASSO regression, namely, age, hospital stay, previous stroke, atrial fibrillation, and NIHSS. Further multivariate logistic regression revealed three independent risk factors affecting patient outcomes, namely, age, previous stroke, and NIHSS. The area under the curve of the nomogram was 0.880, and the 95% confidence interval was 0.818–0.943, suggesting that the nomogram model has good discriminative power. The *p*-value for the calibration curve is 0.925, indicating that the nomogram model is well-calibrated. According to the decision curve analysis results, when the threshold probability is >0.01, the net benefit obtained by the nomogram is the largest. The concordance index for 1,000 bootstrapping calculations is 0.869. The age cutoff for predicting poor patient outcomes using the Youden index was 76.5 years (specificity 0.777 and sensitivity 0.684), the cutoff for the NIHSS was 7.5 (specificity 0.936, sensitivity 0.421), and the cutoff for total nomogram score was 68.8 (sensitivity 81.6% and specificity 79.8%).

**Conclusion:**

The nomogram model established in this study had good discrimination, calibration, and clinical benefits. A nomogram composed of age, previous stroke, and NIHSS might predict the prognosis of stroke after AIS. It might intuitively and individually predict the risk of poor prognosis in 3 months of AIS and provide a reference basis for screening the treatment plan of patients.

## Introduction

Stroke is the most common serious manifestation of cerebrovascular disease and the third leading cause of death in China (1). It mainly causes severe disability, is the leading cause of hospitalization for neurological diseases, and imposes a huge disease burden on China's healthcare system. In the past three decades, the crude stroke mortality rate in China has shown a rapid upward trend, and the growth rate is higher than that of other countries. In the coming years, China will face increasing challenges in reducing stroke morbidity, mortality, and disease burden (2–5).

Severe ischemic stroke has the characteristics of high disability rate, many complications, long hospitalization days, and high mortality rate, which seriously affects the quality of life of patients and brings serious economic and mental burden to patients and their families.

Nomogram's oncology and medical applications are extensive. The nomogram provides individual probabilities of clinical events based on different prognostic and determinant variables, meeting our need for an integrated biological and clinical model that allows us to provide personalized diagnosis and prognosis. Combined with a user-friendly digital interface that improves accuracy and more straightforward prognosis, nomogram prognosis predictions can be seamlessly integrated into clinical decision-making with rapid calculations. Numerous nomograms have appeared on the Internet and in medical journals, and nomograms are increasingly used by patients and physicians (6–8).

In this study, a cohort study was established to collect clinical and laboratory data of hospitalized patients and follow-up with the patients for 3 months to establish a nomogram model for predicting the prognosis of patients with acute ischemic stroke (AIS). We hope to provide a basis for improving clinical decision-making and treatment plans and improve the clinical prognosis of patients.

## Materials and methods

### Patients

The clinically relevant information of patients with AIS who were admitted to the Department of Neurology of Shanghai Jiading District Central Hospital from 18 December 2021 to 8 January 2022 was continuously collected. Inclusion criteria were as follows: (1) diagnosis of AIS with brain magnetic resonance imaging scan during hospitalization to confirm new stroke, (2) age ≥ 18 years, (3) within 7 days after onset, (4) patients or their family members signed the informed consent form and filled in basic information, and (5) expected complete 3-month follow-up. Exclusion criteria were as follows: (1) neoplastic stroke or cerebral venous thrombosis, (2) receiving intravenous thrombolysis or endovascular therapy, and (3) cerebral hemorrhage or transient ischemic attack, or unpredictable psychiatric symptoms. This study was approved by the Jiading District Central Hospital medical ethics committee (No. 2021K04), and the subjects understood, agreed, and signed the informed consent. Patient data are anonymized. The procedures followed were in accordance with the ethical standards of the Jiading District Central Hospital medical ethics committee and the Declaration of Helsinki (1975, revised in 2013).

### Data collection

Demographic characteristics, clinical variables, vascular risk factors, and laboratory results were continuously collected, which included gender, age, length of hospital stay, stroke-related complications (e.g., intracranial or gastrointestinal bleeding, pulmonary infection, urinary tract infection, deep vein thrombosis, and epilepsy), previous history of stroke and atrial fibrillation, years of hypertension and diabetes, alcohol consumption, smoking habits (number of cigarettes smoked per year), blood lipid levels (including triglycerides, high-density lipoprotein, low-density lipoprotein, lipoprotein, and total cholesterol), homocysteine and glycated hemoglobin (HbA1c) levels, carotid intima-media thickness, hematocrit, fibrinogen level on admission, and systolic and diastolic blood pressure on admission. The stroke severity was assessed by the National Institute of Health Stroke Scale (NIHSS) score (9). After 3 months of onset, patient outcomes were obtained through outpatient follow-up or telephone contact and were assessed by modified Rankin scale (mRS) scores (10). An mRS score >3 was defined as a poor outcome (11).

### Statistical analysis

Based on the normality of the distribution, we used the total percentage of categorical variables and the mean ± SD or median and IQR of continuous variables to present baseline patient characteristics in both samples. Differences for numerical and categorical variables were calculated according to Student's *t*-test, Mann-Whitney *U* test, Pearson's χ test, or Fisher's exact test, as appropriate.

The least absolute shrinkage and selection operator (LASSO) regression technique (12) is used for data dimension and predictor selection. Multivariate logistic regression-based analysis was used to develop predictive models and draw a nomogram for poor prognosis. The nomogram scores the value of each variable according to the size of the regression coefficient. The total score for each patient is calculated, and then the probability of an outcome for each patient can be calculated using a conversion function between the score and the probability. The area under the curve (AUC) of the receiver operating characteristic curve (ROC) analysis and concordance index (C-index) were used to assess the accuracy of the nomogram in predicting patient outcomes. Based on the calibration curve, we evaluated the agreement between predicted and observed probabilities. By quantifying the net benefit across all threshold probabilities, the decision curve analysis (DCA) was carried out to evaluate the clinical usefulness of nomograms (13, 14).

The Youden index was used to calculate the optimal threshold of the nomogram for predicting stroke prognosis, and according to the optimal threshold, all patients were further divided into good and poor prognosis groups. The bootstrap method (resampling = 1,000) is used for internal validation.

Statistical analysis was performed using the R software (version 4.1.2, https://www.r-project.org). The nomogram was generated using the “rms” package of R software. Two-way statistical significance levels are reported, with *p* < 0.05 considered statistically significant.

## Results

A total of 146 patients were enrolled in this study, of whom eight were subsequently lost to follow-up, six were excluded for endovascular intervention due to disease progression, and 132 were eventually enrolled. Good outcome was found in 94 (71.212%) patients and bad outcome in 38 (28.788%) patients during the follow-up period. A total of eight (6.061%) deaths were reported over this period. Of whom, five (3.788%) died during hospitalization (two patients died due to progressive ischemic stroke and three due to pulmonary complications) and three were discharged from the hospital, of whom one patient had colonic cancer, one patient had lung cancer, and two patients died of cardiac-related deaths at the 3-month follow-up.

**Figure 1:**
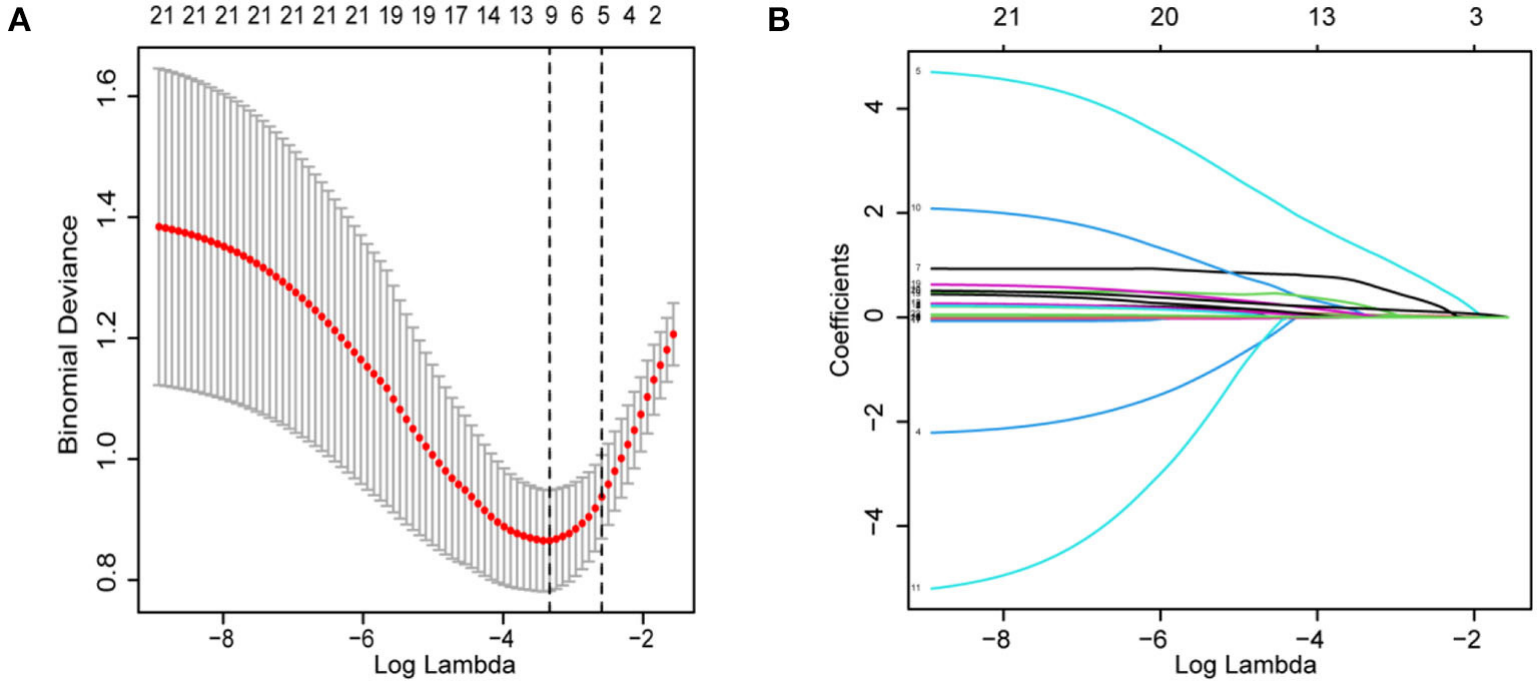
Demographics, clinical characteristics, vascular risk factors, and laboratory outcomes were selected using the LASSO binary logistic regression model. **(A)** In LASSO regression, the adjustment parameter (lambda) for bias was chosen based on the minimum criterion (left dashed line) and the 1-SE criterion (right dashed line). The lambda value is 0.0750. **(B)** Coefficient profiles were created from the log(lambda) series. In this study, the choice of predictors was based on the 1-SE criterion (right dashed line). Among the best results are features with five nonzero coefficients. LASSO, least absolute shrinkage and selection operator; SE, standard error.

The patient characteristics in the cohort are shown in Table 1. Subsequently, we performed LASSO regression to select highly correlated variables from the baseline characteristics of the cohort patients, resulting in lambda.min = 0.036 [log(lambda.min) = −3.335] and lambda.1se = 0.075 [log(lambda.1se) = −2.591]. Considering the small sample size, lambda.1se was chosen. When lambda.1se = 0.075, variables were filtered including age, length of hospital stay, previous history of stroke and atrial fibrillation, and NIHSS (Figure 1).

**Table 1:** Baseline characteristics of cohort patients.

	**Level**	**Overall**
n		132
Gender (%)	Female	47 (35.6)
	Male	85 (64.4)
Age (years, median [IQR])		69.00 [60.00, 81.00]
Length of hospital stay (days, median [IQR])		11.00 [8.75, 13.00]
Stroke-related complications (%)	No	116 (87.9)
	Yes	16 (12.1)
History of stroke (%)	No	90 (68.2)
	Yes	42 (31.8)
Years of hypertension (median[IQR])		8.00 [0.00, 22.00]
AF(%)	No	117 (88.6)
	Yes	15 (11.4)
Years of diabetes (median [IQR])		0.00 [0.00, 3.00]
Number of cigarettes smoked per year (median [IQR])		0.00 [0.00, 600.00]
Alcohol consumption (%)	No	104 (78.8)
	Yes	28 (21.2)
IMT (mm, median [IQR])		0.94 [0.90, 1.00]
HCT (%, median [IQR])		39.76 [37.10, 43.42]
Fg (g/dl, median [IQR])		2.96 [2.50, 3.34]
HCY (μmol/L, median [IQR])		15.30 [12.30, 17.40]
TG (mmol/L, median [IQR])		1.36 [1.06, 1.90]
LDL (mmol/L, median [IQR])		3.11 [2.65, 3.51]
HDL (mmol/L, median [IQR])		1.01 [0.89, 1.22]
TC (mmol/L, median [IQR])		5.65 [4.90, 6.49]
HbA1C (%,median [IQR])		6.20 [5.70, 6.89]
NIHSS (median [IQR])		3.00 [2.00, 5.00]
SBP (mmHg, mean (SD))		154.16 (21.01)
DBP (mmHg, median [IQR])		87.00 [82.00, 96.50]
mRS (%)	0	94 (71.2)
	1	38 (28.8)

AF, history of atrial fibrillation; IMT, carotid intima-media thickness; HCT, hematocrit; Fg, fibrinogen; HCY, homocysteine; TG, triglycerides; LDL, low-density lipoprotein; HDL, high-density lipoprotein; TC, total cholesterol; HbA1C, glycosylated hemoglobin, Type A1C; NIHSS, the National Institute of Health Stroke Scale score; SBP, systolic blood pressure; DBP, diastolic blood pressure; mRS, modified Rankin scale scores.

To establish a predictive nomogram model for poor prognosis of AIS, multivariate logistic regression analysis was performed based on the above five variables selected by the LASSO regression technique, and the results showed that age (odds ratio (OR), 1.076 [95% confifidence interval [CI], 1.029 to 1.130]), previous stroke (OR, 7.441 [95% CI, 2.567 to 23.860]), and NIHSS (OR, 1.271 [95% CI, 1.109 to 1.500]) were identified as independent predictors (Table 2).

**Table 2:** Multivariate logistic regression analysis variables screened.

	**β**	**Odds ratio**	**95%CI**	**P value**
Intercept	−9.479	0.000	(0.000, 0.003)	0.000^*^
Age(years)	0.073	1.076	(1.029, 1.130)	0.002^*^
Length of hospital stay(days)	0.108	1.114	(0.994, 1.281)	0.114
Previous stroke	2.007	7.441	(2.567, 23.860)	0.000^*^
AF	0.816	2.263	(0.548, 10.038)	0.261
NIHSS	0.240	1.271	(1.109, 1.500)	0.002^*^

β is the regression coefficient. The prognosis of patients was divided into good prognosis and poor prognosis using the mRS score and entering the logistic regression model.

AF, history of atrial fibrillation; NIHSS, the National Institute of Health Stroke Scale score.

* :< 0.05.

**Figure 2:**
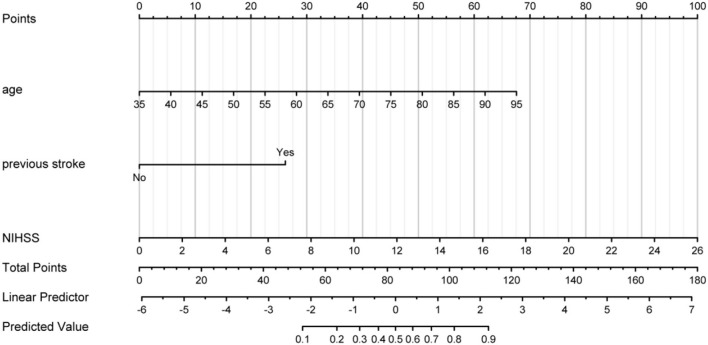
Prognostic nomogram developed for AIS. The nomogram included age, previous stroke, and NIHSS. The nomogram and its algorithm used to predict the risk of poor prognosis in AIS are as follows. First, find the corresponding score on the points line at the top of each variable for patients with AIS; then add all the scores and find the corresponding point on the total points. Finally, find the predicted probability corresponding to the patient on the predicted value line. AIS, acute ischemic stroke; NIHSS, the National Institute of Health Stroke Scale score.

A predictive nomogram model was developed based on the above independent predictors and presented as a nomogram (Figure 2).

**Figure 3:**
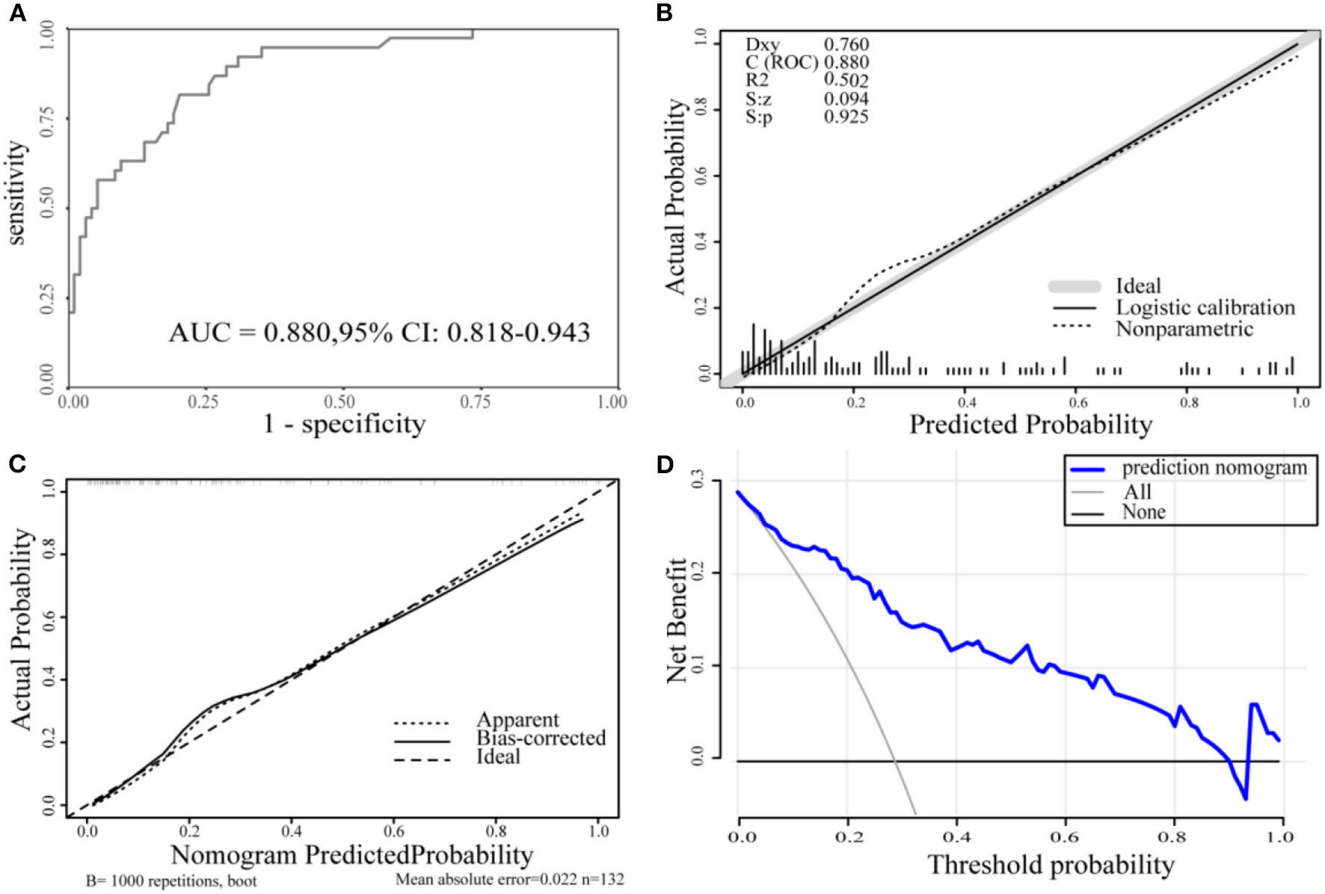
**(A)** The ROC curve for the nomogram. AUC = 0.880 (95% CI, 0.818-0.943). **(B)** The calibration curve for the nomogram. *p* = 0.925 > 0.05. **(C)** The calibration curve for the nomogram with 1,000 bootstrap resamples. **(D)** DCA of the nomogram. The solid blue line represents the nomogram. Decision curves show that when the threshold probability is >0.01, the nomogram achieves the greatest benefit compared with all treatment and no treatment strategies. ROC, the receiver operating characteristic curve; AUC, the area under the curve; DCA, decision curve analysis.

The AUC for the predictive nomogram model was 0.880 (95% CI confidence interval [CI], 0.818–0.943) (Figure 3A). The C-index for the nomogram model is 0.880. The internal validation C-index using bootstrapping (resampling = 1,000) is 0.869. The *p*-value calculated from the calibration curve of the nomogram in AIS was 0.925, indicating that the data were unbiased and a perfect fit (Figure 3B). A calibration curve drawn using 1,000 resampling for bootstrapping validation showed good agreement between predictions and observations as well (Figure 3C). Finally, decision-curve analysis (DCA) was performed to assess whether this nomogram could help distinguish patients with AIS between good and poor prognoses (Figure 3D). According to the DCA results, when the threshold probability is >0.01, the net benefit obtained by the nomogram is the largest. The Youden index was used to select the cutoff point for reclassification. The cutoff points for predicting patient outcomes using age and NIHSS alone were 76.5 years (specificity, 0.777; sensitivity, 0.684) and 7.5 (specificity, 0.936; sensitivity, 0.421), respectively. The cutoff point for the total points of the nomogram in the cohort was 68.8 (sensitivity, 81.6%; specificity, 79.8%).

## Discussion

We developed and validated a predictive nomogram based on demographic characteristics and clinical factors for individualized prediction of outcome 3 months after AIS onset. Although the poor prognosis of AIS is mainly due to the severity of the disease, an accurate prognosis is critical for many clinical decisions in patients with AIS. To avoid the disadvantages of overfitting and skewed distribution of predictors using traditional logistic regression analysis, LASSO regression was used to analyze demographics, vital signs, comorbidities, and laboratory variables. The predicted outcome associations were examined by narrowing down the regression coefficients using the LASSO method, reducing the 22 candidate feature variables to five potential predictors. Based on multivariate logistic regression analysis, independent influencing factors were screened, and nomograms were drawn. The nomogram included three variables, including age, previous stroke, and NIHSS, and showed good discriminatory power, calibration, and clinical utility.

Many variables have been reported to be associated with AIS prognoses, such as age, gender (15), NIHSS (16), atrial fibrillation (17), and previous stroke (18).

Age is an independent and uncontrollable risk factor for ischemic stroke (15). Statistics from the American Heart Association show that the prevalence of stroke increases with age (19, 20). The cardiovascular risk factors of ischemic stroke are different in patients of different ages. Several risk factors such as smoking and hyperlipidemia were attributed to young adults (21). Prospective cohort studies have found different cardiovascular risk factors in patients aged above 80 years (22, 23). For example, females, atrial fibrillation, and hypertension were reported more frequently in patients 80 years and older, while congestive heart failure, kidney disease, and prior cerebral infarction were reported more frequently in patients 85 years and older. Our study found that age (OR, 1.076 [95% CI, 1.029 to 1.130]) was an independent risk factor for poor prognosis in AIS when patients were older than 76.5 years. This is consistent with previous reports (22, 23). At the same time, we found that the age of poor prognosis of patients in this study was smaller than that in previous studies, whose one explanation could be based on the fact that patients have an earlier age of chronic diseases and the onset of complications such as heart failure, atrial fibrillation, and kidney failure, with the increase of living standards.

Many literatures suggest that gender is associated with prognosis in AIS (15, 24–26). The incidence of ischemic stroke in women increases with the onset of menopause after the age of 42 years (27). In patients older than 80 years, female patients were more likely to have an ischemic stroke [OR 2.27 (95 CI%: 1.71–3.02)] (22), whose possible mechanism is that estrogen is a protective agent for ischemic stroke (28). However, our study showed that gender is not an independent risk factor for poor prognosis in patients with AIS. Results of a door-to-door survey based on a nationally representative sample showed no statistically significant gender differences in age-standardized stroke incidence (3). A Chinese population-based clinical intervention cohort study found no statistical difference between genders after intravenous thrombolysis in AIS (29), which is consistent with our results (29). This result requires further confirmation by subgroup analysis, and the possibility of ethnic differences cannot be ruled out.

As a tool to assess the severity of neurological deficits in patients, NIHSS can also predict the prognosis of patients. Generally, the higher the NIHSS, the worse the prognosis of patients (16, 30). This study found that the patient's NIHSS (OR, 1.271 [95% CI, 1.109–1.500]) was an independent risk factor for evaluating the prognosis of patients. When the NIHSS was >7.5, the risk of poor prognosis was higher.

Atrial fibrillation can form a cardioembolic thrombus to block intracranial blood vessels and cause stroke. At present, many studies believe that atrial fibrillation is a risk factor for AIS (17, 20). In this study, atrial fibrillation was found to be associated with prognosis in patients with AIS by LASSO regression analysis. However, further multivariate logistic regression did not find that atrial fibrillation was an independent risk factor for the prognosis of patients with AIS. This may require further validation with a larger sample size of participants and longer follow-up.

Patients with previous stroke have more severe intracranial vascular stenosis and worse collateral circulation compensation, which has an important impact on the prognosis of AIS, and many literature reports have confirmed this conclusion (31, 32). The results, in this study, show that previous stroke (18, 20) (OR, 7.441 [95% CI, 2.567 to 23.860]) is an independent risk factor for prognosis in patients with AIS. In this nomogram model, the OR value of a previous stroke was significantly higher than that of other stroke, suggesting that it has an important impact on patient prognosis. We speculated that as the number of previous stroke increases, the prognosis of patients may be worse, which needs to be further verified in follow-up studies.

At present, the scales commonly used to evaluate the prognosis of patients are the ischemic stroke predictive risk score (iScore) and the acute stroke registry and analysis of Lausanne (ASTRAL) score (33).

The iScore's items included age, sex, stroke severity, stroke subtype, risk factors, comorbidities, prehospital disability, and blood glucose on admission and were primarily used to predict a patient's 30-day and 1-year mortality risk. The 30-day and 1-year mortality risks of patients were predicted by the iScore model with a C-index of 0.850 and 0.823, respectively, and validated with internal and external datasets (34). ASTRAL's items included age, severity, time delay from onset to admission >3 h, degree of visual field deficit, acute glycemia, and decreased level of consciousness. The AUC for the score in the ASTRAL (33) cohort was 0.850, well-calibrated in derivation (*p* = 0.43). Verified by external data, the AUC is high, and the calibration is good.

The nomogram in this study showed sufficient discrimination (AUC, 0.880, 95% CI, 0.818–0.9343; C-index, 0.880), and then internal validation with bootstrap resampling also yielded satisfactory results (C-index, 0.869). The *p*-value of the calibration curve is 0.925, indicating that the nomogram model has a high degree of calibration and a good fit. Internal verification of its calibration again yielded a consistent conclusion.

Decision curve analysis showed that when the threshold probability was >0.01, the nomogram had a higher clinical application value and better clinical practicability. Compared with the above two models, the model in this study has fewer evaluation items, and the patient data information is easy to collect. Further evaluation found that the model has better accuracy, calibration, and clinical efficacy. However, the sample size for establishing the model is relatively small, and it is necessary to further expand the sample and external validation and to compare the models through rigorous statistical methods.

At the same time, to increase the practicability of the model and facilitate the calculation, we calculated the cutoff point of the nomogram to distinguish between patients with good prognosis and poor prognosis. When the total score was >68.8, the patient was considered to have a poor prognosis at 3 months.

This study has some limitations. First, whether a patient can accept hospitalization in the neurology department of our hospital is affected by many factors, including the judgment of the clinician, the economic level of the patient, and the number of beds in the neurology department of our hospital, which may cause selection bias. Second, as a single-center study, the population is relatively small. Although our robustness nomogram was extensively tested using a bootstrapped internal validation test, further studies with more patients and external multicenter data are needed to further validate our findings. Third, due to the recent outbreak of novel coronavirus infection in Shanghai, it may have an impact on the prognosis of a few patients who were followed up. Fourth, this study does not involve the influence of intravenous thrombolysis, endovascular interventional therapy, and other treatment options on prognosis, which needs further study. Fifth, AIS subtypes were not included in this study. Further subgroup analysis of AIS may yield more specific results. In particular, lacunar ischemic stroke is a common type of stroke and the first clinical manifestation of small vascular disease. In China, small vascular disease due to arteriolar occlusion accounts for approximately 30% of ischemic stroke causes (4). However, the diagnosis and transformation of the small vascular disease still need further research (35). In addition, we found relatively few clinical prediction models for small vascular disease, which will be the direction of further research in the future. Finally, our nomogram model may only be useful for rapidly identifying risk for poor prognosis 3 months after onset, without providing information on possible pathophysiological mechanisms. Despite these limitations, this study developed a simple, valid, and personalized nomogram for predicting the risk of a poor 3-month prognosis in AIS.

## Conclusion

In this study, combining three demographic and clinical characteristics, a nomogram was developed to predict the 3-month prognostic risk of AIS. The nomogram has high discriminative power, good calibration, and a wide net gain threshold range. Patients were considered to have a poor prognosis when the nomogram score was greater than 68.8. The tool involves three collected variables and can be easily used at the bedside. This may be a useful addition to current AIS prognostic assessments, providing a basis for clinicians to make better clinical treatment decisions and early rehabilitation assessment protocols for patients.

## Data availability statement

The raw data supporting the conclusions of this article will be made available by the authors, without undue reservation.

## Ethics statement

The studies involving human participants were reviewed and approved by the Jiading District Central Hospital Medical Ethics Committee. The patients/participants provided their written informed consent to participate in this study.

## Author contributions

Conception and design: GD and ZN. Collection and assembly of data and data analysis and interpretation: GD, JX, JH, and ZN. Manuscript writing and final approval of manuscript: All authors.

## Funding

This study was funded by the Science and Technology Commission of Jiading District (Grant No. JDKW-2019-W29), China.

## Conflict of interest

The authors declare that the research was conducted in the absence of any commercial or financial relationships that could be construed as a potential conflict of interest.

## Publisher's note

All claims expressed in this article are solely those of the authors and do not necessarily represent those of their affiliated organizations, or those of the publisher, the editors and the reviewers. Any product that may be evaluated in this article, or claim that may be made by its manufacturer, is not guaranteed or endorsed by the publisher.
